# Synthesis and characterization of selenium nanoparticles stabilized with cocamidopropyl betaine

**DOI:** 10.1038/s41598-022-25884-x

**Published:** 2022-12-20

**Authors:** Andrey V. Blinov, Andrey A. Nagdalian, Shahida A. Siddiqui, David G. Maglakelidze, Alexey A. Gvozdenko, Anastasiya A. Blinova, Mariya A. Yasnaya, Alexey B. Golik, Maksim B. Rebezov, Seid Mahdi Jafari, Mohd Asif Shah

**Affiliations:** 1 North-Caucasus Federal University, Pushkina Str. 1, Stavropol, Russia 355017; 2 Campus Straubing for Biotechnology and Sustainability, Technical University of Munich (TUM), Essigberg 3, 94315 Straubing, Germany; 3 German Institute of Food Technologies (DIL e.V.), Prof.-von-Klitzing-Straße 7, 49610 Quakenbrück, Germany; 4 Russian State Agrarian University - Moscow Timiryazev Agricultural Academy, Moscow, Russia; 5 Department of Scientific Research, V. M. Gorbatov Federal Research Center for Food Systems, Moscow, Russia; 6 Department of Food Materials and Process Design Engineering, Gorgan University of Agricultural Science and Natural Resources, Gorgan, Iran; 7 Nutrition and Bromatology Group, Department of Analytical Chemistry and Food Science, Faculty of Science, University of Vigo, 32004 Ourense, Spain; 8 Department of Economics, Kebridehar University, Kebri Dehar, Somali Post Box 250, Ethiopia; 9 Adjunct Faculty, School of Business, Woxsen University, Hyderabad, Telangana 502345 India

**Keywords:** Nanoparticles, Chemical engineering, Chemical engineering

## Abstract

In this work, selenium nanoparticles (Se NPs) stabilized with cocamidopropyl betaine were synthesized for the first time. It was observed that Se NPs synthesized in excess of selenic acid had a negative charge with ζ-potential of −21.86 mV, and in excess of cocamidopropyl betaine—a positive charge with ξ =  + 22.71 mV. The resulting Se NPs with positive and negative charges had a spherical shape with an average size of about 20–30 nm and 40–50 nm, respectively. According to the data of TEM, HAADF-TEM using EDS, IR spectroscopy and quantum chemical modeling, positively charged selenium nanoparticles have a cocamidopropylbetaine shell while the potential- forming layer of negatively charged selenium nanoparticles is formed by SeO_3_^2−^ ions. The influence of various ions on the sol stability of Se NPs showed that SO_4_^2−^ and PO_4_^3−^ ions had an effect on the positive Se NPs, and Ba^2+^ and Fe^3+^ ions had an effect on negative Se NPs, which corresponded with the Schulze-Hardy rule. The mechanism of coagulating action of various ions on positive and negative Se NPs was also presented. Also, influence of the active acidity of the medium on the stability of Se NPs solutions was investigated. Positive and negative sols of Se NPs had high levels of stability in the considered range of active acidity of the medium in the range of 1.21–11.98. Stability of synthesized Se NPs stability has been confirmed in real system (liquid soap). An experiment with the addition of Se NPs stabilized with cocamidopropyl betaine to liquid soap showed that the particles of dispersed phases retain their initial distributions, which revealed the stability of synthesized Se NPs.

## Introduction

As a result of self-organization and self-assembly, supramolecular structures of various morphologies can be formed, e.g., nanostructures of the "core–shell" type^1^. Nanosystems based on the biogenic element selenium are of particular interest. Selenium nanoparticles (Se NPs) are already being used as highly sensitive biosensors for immunoassay and chromatographically mobile affinity reagents^2–4^. Even at very low concentrations of Se NPs in water (0.005–0.1%), these nanoparticles can adsorb antigens and antibodies on their surface^5–7^. Se NPs can stimulate the germination of crop seeds^8,9^.It is known that Se NPs, which are a part of food products, have an antiblastic effect^10,11^, and there is an inversely proportional relationship between the selenium content in the external environment and the incidence of malignant tumors in the population^12,13^. Se NPs have high antitumor and biological activity, due to which they participate in the regulation of the formation of antioxidants and prevent the growth and development of cancer cells^14–16^. It is also known that in conditions of selenium deficiency, the development of myocardiodystrophy, atherosclerosis, coronary heart disease, myocardial infarction, chronic hepatitis of various etymologies and viral infections is observed^17–21^.

One of the actual directions of Se NPs’ research is stabilization in the nanoscale state. Work of many authors on the stabilization of Se NPs are based on the use of polysaccharides as well as various polymers, ionic and nonionic surfactants^22–29^. Known methods of stabilizing Se NPs in aqueous medium using polymers have a common disadvantage. The polymeric matrix most often does not provide the necessary aggregate stability of the system due to the hydrophobic nature of selenium. Achieving high aggregate stability of the system is accompanied by a decrease in the activity of Se NPs^5,30–32^.

For the qualitative stabilization of Se NPs, it is necessary to use surfactants having both hydrophobic and hydrophilic components. Under certain physicochemical conditions, such surfactants, when interacting with hydrophobic Se NPs, can change their hydrophobic surface nature to hydrophilic, and hydrophilic colloids are known to be much more stable in aqueous media^33–36^. Currently, one of the industrially most important amphiphilic surfactants is cocamidopropyl betaine (CAPB)^37^. The widespread use of CAPB in the industry is due to its antiseptic properties, as well as its ability to act as a surfactant, thickener and emulsifier^38,39^.

We considered that the synthesis of CAPB-stabilized Se NPs has prospects for creating a stable molecular system. As far as we know, this is the first attempt to stabilize Se NPs with CAPB, which determines the scientific novelty of this work. Therefore, the purpose of this work was the synthesis of Se NPs stabilized with CAPB and evaluating the aggregate stability of created nanosystem in a wide pH range, as well as, using solutions with various ions and model samples of liquid soap.

## Materials and methods

Reagent grade chemicals and grade A glassware were used in the present study. Conductivity of distilled water used was < 1 µS/cm.

### Synthesis of Se NPs stabilized with CAPB

Synthesis of Se NPs stabilized with CAPB (*Matrix Oleochem Sdn Bhd, Egypt*) was carried out by chemical reduction in an aqueous medium. Selenic acid (*Lenreactive, Russia*) was used as a selenium-containing precursor, and ascorbic acid (*Lenreactive, Russia*) was used as a reducing agent. Samples with positively and negatively charged Se NPs were obtained for the next steps.

#### Synthesis of the positive Se NPs sol

At the first stage, selenic acid (m = 470 mg) and CAPB (m = 5240 mg) were weighed by precision balances ML203T/A00 (*Mettler Toledo, Russia*) and dissolved in 100 mL distilled water. A solution of ascorbic acid was then prepared by dissolving 773.8 mg ascorbic acid in 50 mL distilled water. At the last stage of synthesis, with intensive stirring, an ascorbic acid solution was simultaneously poured into a solution with a precursor and stabilizer, and the resulting red sol (highly dispersed colloidal system of Se NPs) was mixed for 5–10 min at 500 rpm using multi mixer MM1000 (*Biosan, Latvia*). The Convergence electrodialysis laboratory unit (*Convergence, Twente, Netherlands*) was used to remove the reaction by-products.

#### Synthesis of the negative Se NPs sol

First, selenic acid (m = 3560 mg) and CAPB (m = 680 mg) were weighed by precision balances ML203T/A00 (*Mettler Toledo, Russia*) and dissolved in 100 mL distilled water. A solution of ascorbic acid was then prepared by dissolving 773.8 mg ascorbic acid in 50 mL distilled water. At the last stage of synthesis, with intensive stirring, an ascorbic acid solution was simultaneously poured into a solution with a precursor and stabilizer, and the resulting red sol was mixed for 5–10 min at 500 rpm using multi mixer MM1000 (*Biosan, Latvia*).

### Characterization of synthesized Se NPs

The microstructure of Se NPs was studied using a transmission electron microscope (TEM) Carl Zeiss Libra 120 M (*Carl Zeiss AG, Germany*). Se NPs were applied by ultrasonic dispersion of a prepared solution and water in a ratio of 1:1 on copper grids with a carbon base. The magnitude of the accelerating voltage was 120 kV.

The determination of the average hydrodynamic radius of the particles was carried out by the dynamic light scattering (DLS) method on a Photocor-Complex instrument (*Antek-97, Russia*). Processing of the results was carried out using the DynaLS software (*Antek-97, Russia*).

Measurement parameters:

- measuring angle −90°
- solvent–water
- number of measurements per cycle −100.

The ζ-potential was determined by acoustic and electroacoustic spectroscopy on a DT-1202 setup (*Dispersion Technology Inc., USA*).

The molecular simulation was carried out in the IQmol molecular editor (*Q-Chem, USA*), the quantum-chemical calculations of the models were carried out using the QChem software (*Q-Chem, USA*) with the following parameters: Calculation—Energy, method—B3LYP, Basis—6-31G*, Convergence—4, Force field – Chemical. All studies were carried out in five-fold repetition. The significance of the experimental results was determined using the Fisher criterion.

To study the functional groups in the obtained samples, IR spectroscopy was used. IR spectra were recorded on an FSM-1201 IR spectrometer with Fourier transform. The measurement range was 500–4000 cm^−1^.

X-Ray diffraction analysis (XRD) was conducted using X-Ray diffractometer Empyrean (*PANalytical, Almelo, The Netherlands*) to determine the crystalline structure of nanoparticles. Preparation of samples for X-ray diffraction analysis included the addition of selenium sol to aerosil powder (*Lenreactive, Russia*) and homogenization of the powder containing selenium. Measurement parameters:

- Copper cathode
- Emission wavelength: 1.54 A
- Current: 35 mA
- Voltage: 40 kV
- 2θ measurement range: 10–90◦
- 2θ sampling frequency: 0.01◦.

High-angle annular dark-field scanning transmission electron microscopy (HAADF-TEM) with energy-dispersive X-ray spectroscopy (EDS) was performed on a high-resolution transmission electron microscope JEOL "JEM-2200FS CS" (*JEOL, Tokyo, Japan*). For research, the sample was mixed 1:1 with distilled water and applied to a copper mesh with a carbon substrate.

Measurement parameters:

- Accelerating electron beam voltage −200 kV
- Spot size (EDS) −0.5 nm
-The accumulation time of one spectrum −300 s

### The effect of various ions on the stability of Se NPs

To study the effect of ions on the stability of Se NPs, the following salts were selected: NaCl (*Lenreactive, Russia*), Na_2_SO_4_ (*Lenreactive, Russia*), Na_3_PO_4_ (*Lenreactive, Russia*), BaCl_2_ (*Lenreactive, Russia*), FeCl_3_ (*Lenreactive, Russia*). Samples of positive and negative Se NPs sols in a volume of 30 mL were prepared. The positive sol of Se NPs was taken into 5 tubes of 5 mL, then NaCl, Na_2_SO_4_, Na_3_PO_4_, FeCl_3_ and BaCl_2_ salts with concentrations 0.1 M, 0.25 M, 0.5 M, 0.75 M and 1 M were added to them. For the remaining salts, the process was repeated according to the above procedure. Similar manipulations were performed for the negative sol of Se NPs.

### The effect of pH on the stability of Se NPs

To determine the effect of active acidity of medium on the stability of Se NPs, samples of positive and negative Se NPs sols in a volume of 100 mL and 0.1 M HCl and NaOH solutions from standard titers (*Ekroschem, Russia*) were prepared. Positive and negative Se NPs sols were divided into 11 samples and 0.1 M HCl and NaOH solutions were added to each to create a certain value of the active acidity of the medium from 2.21 to 11.98. The active acidity of the medium was measured using a pH meter (ionomer) Expert-001 (*Econix-Expert, Moscow, Russia*) with a combined pH electrode ESC-10605/7 with a thermal sensor.

### The stability of Se NPs in model samples of liquid soap

Se NPs stabilized with CAPB have a great potential of industrial application, that is why determination of stability of synthesized nanoparticles was very important in our work. For this, we chose a liquid soap as real system which contains many substances that can cause coagulation of Se NPs.

To study the stability of Se NPs in experimental samples of liquid soap, samples of positive and negative Se NPs sols in a volume of 10 mL and liquid soap in a volume of 100 mL were prepared. The synthesis of the liquid soap was carried out as follows:

In 50 mL distilled water, 1 g of NaOH (*Povolzhe, Russia*) was dissolved. Then 1.8 g oxy-ethylidenediphosphonic acid (OEDP) (*HimTEK, Russia*) and 0.5 g disodium salt of ethylenediaminetetraacetic acid (EDTA) (*Mosreactive, Russia*) was added sequentially. In the prepared mixture, 6 g sodium lauryl ether sulfate (*HimEtalon-NN, Russia*) and 8 g CAPB was dissolved. After complete dissolution, 1 g NaCl (*Lenreactive, Russia*) and 50 mL distilled water were added sequentially. When mixing Se NPs sols with prepared liquid soap, a ratio of 1:99 was used. Determination of the average hydrodynamic radius of particles in the obtained liquid soap samples with positive and negative Se NPs sols was carried out by the DLS method on a Photocor-Complex instrument (*Antek-97, Russia*), using the DynaLS software (*Antek-97, Russia*).

The surface tension was determined on an automatic DCAT tensiometer (*DataPhysics Instruments GmbH, Filderstadt, Germany*).

Viscometry was performed on Fungilab Expert rotary viscometer (*Fungilab S. A,, Madrid, Spain*), the action of which is based on the use of viscous friction arising in a layer of liquid flowing in an annular gap between a rotating and stationary cylinder. Parameters of the measurement:

- Cylinder TL: 1–8
- Temperature: 25 °C
- Rotation speed: 0.1–200 rpm.

The electrical conductivity was determined using Expert 001 pH meter–ionomer (*Econix-Expert, Moscow, Russia*) using a platinum electrode (EPV-1 cp).

The refractive index was determined using IRF-454b2m refractometer (*KOMZ, Moscow, Russia*).

## Results and discussion

### Results of the characterization of synthesized Se NPs

**Figure 1:**
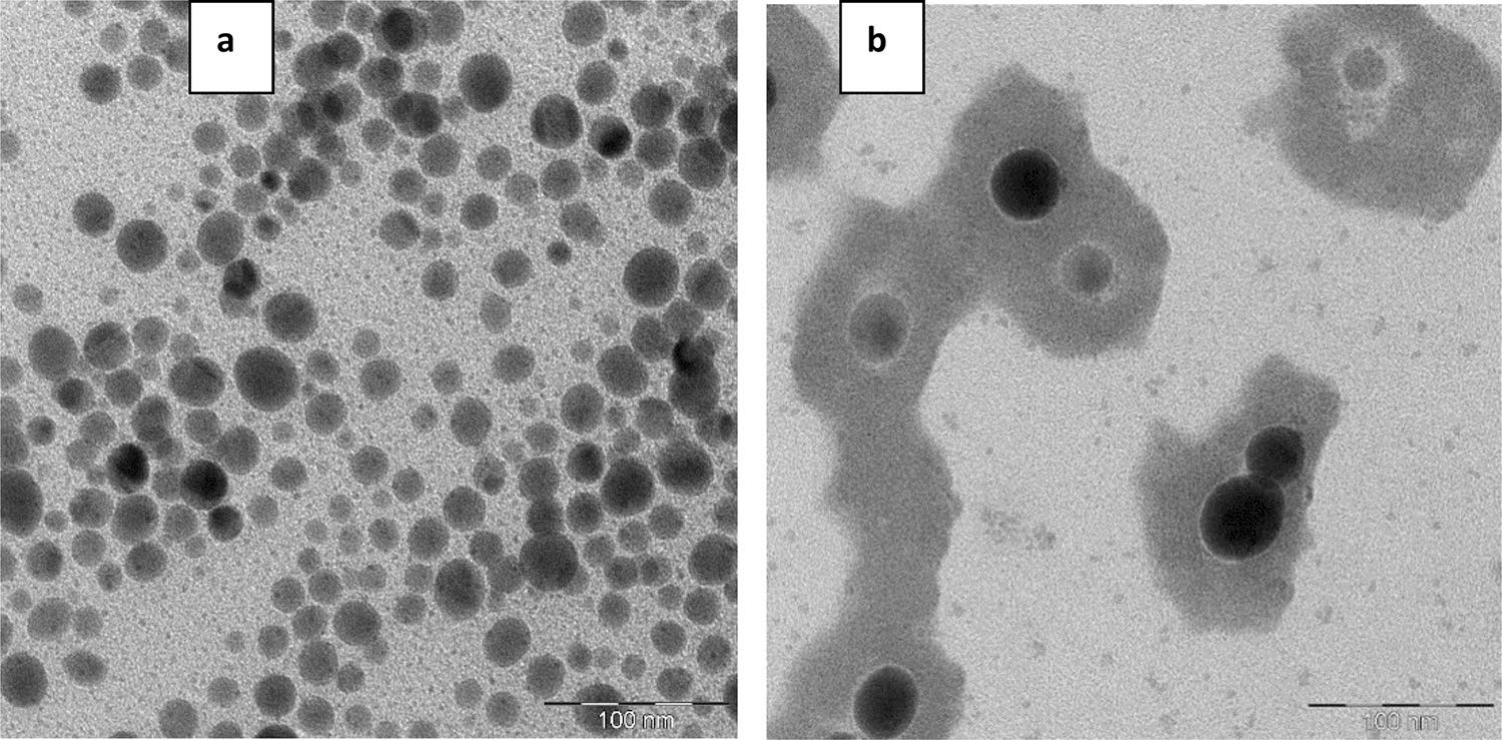
Transmission electron microscopy of Se NPs samples at magnification × 640000: (**a**) the negative sol, (**b**) the positive sol.

**Figure 2:**
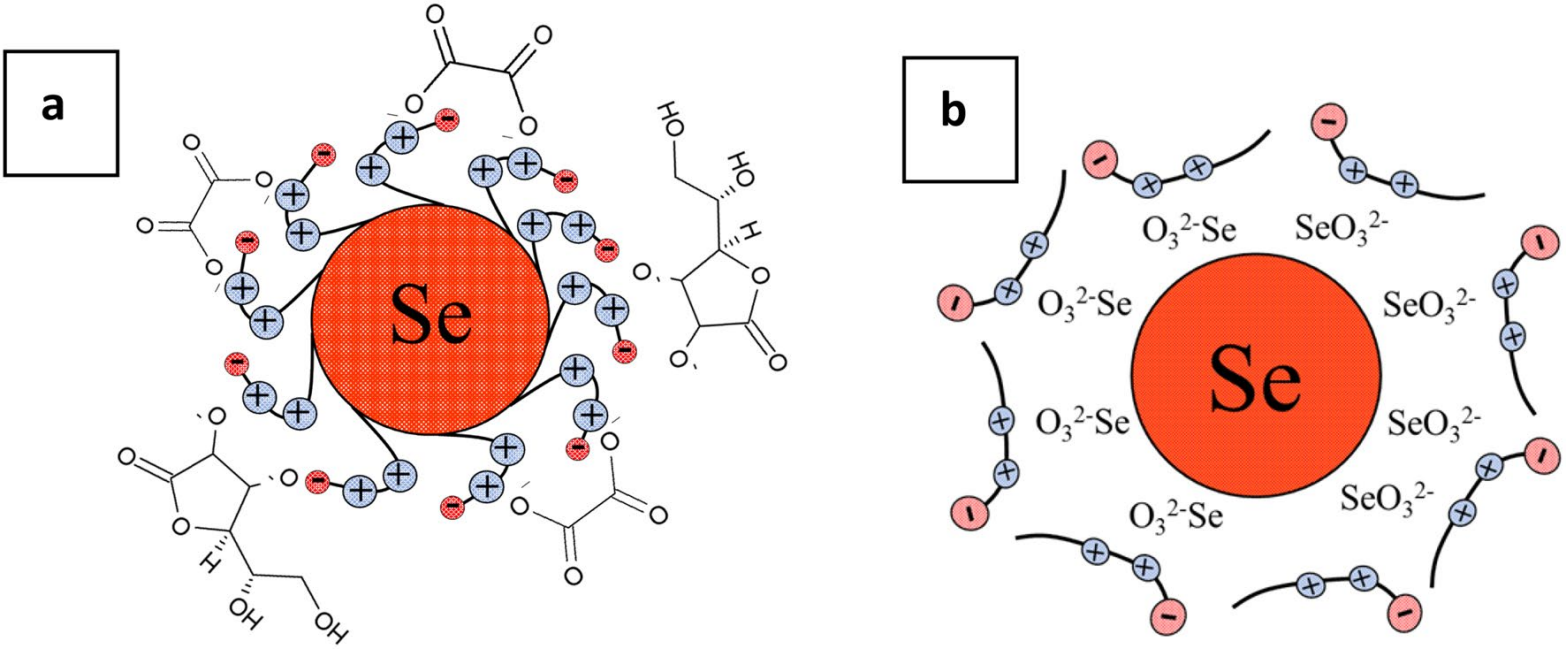
Structure of micelles of Se NPs stabilized with CAPB: (**a**) the positive sol micelle; (**b**) the negative sol micelle.

At the first stage, the ζ-potential of obtained Se NPs was determined. It was found that the ζ-potential was + 22.71 ± 2.14 mV in the positive Se NPs sol and −21.86 ± 1.87 mV in the negative Se NPs sol. As a result of the analysis of TEM images (Fig. 1), Se NPs with both positive and negative charges had a spherical shape with an average size of about 20–30 nm and 40–50 nm, respectively, which corresponds to the data of other authors^22,25,40^. It is important to note the distinctive feature of the obtained nanoparticles—The presence of a layer of a surfactant compound (CAPB) on the surface of the positive Se NPs. Based on TEM results, the structure of micelles of Se NPs stabilized with CAPB are shown schematically in Fig. 2.

**Figure 3:**
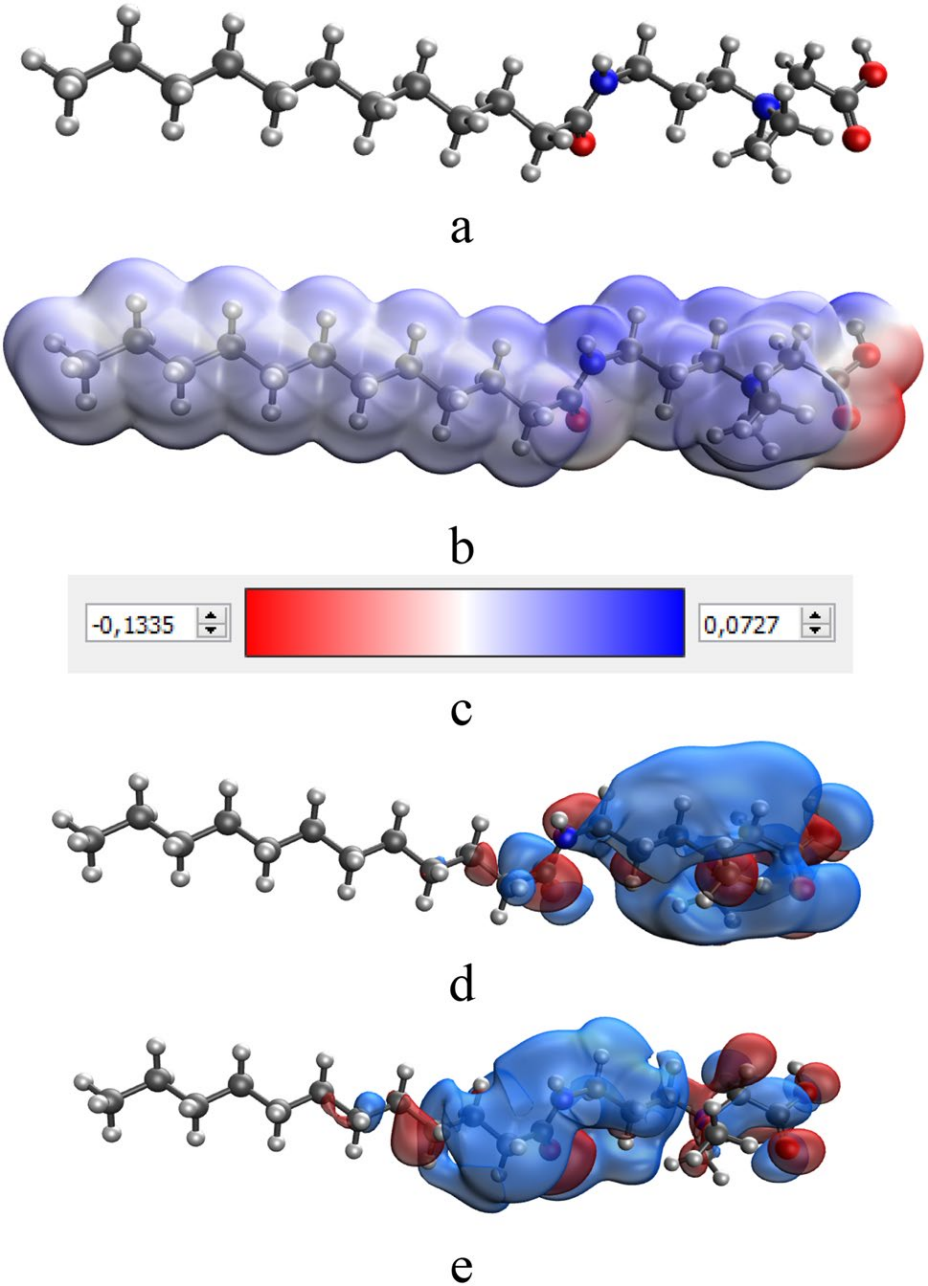
Quantum chemical modeling of CAPB molecule: (**a**) model of the molecule, (**b**) electron density distribution, (**c**) electron density distribution gradient, (**d**) the highest occupied molecular orbital (HOMO), (**e**) the lowest occupied molecular orbital (LUMO).

**Figure 4:**
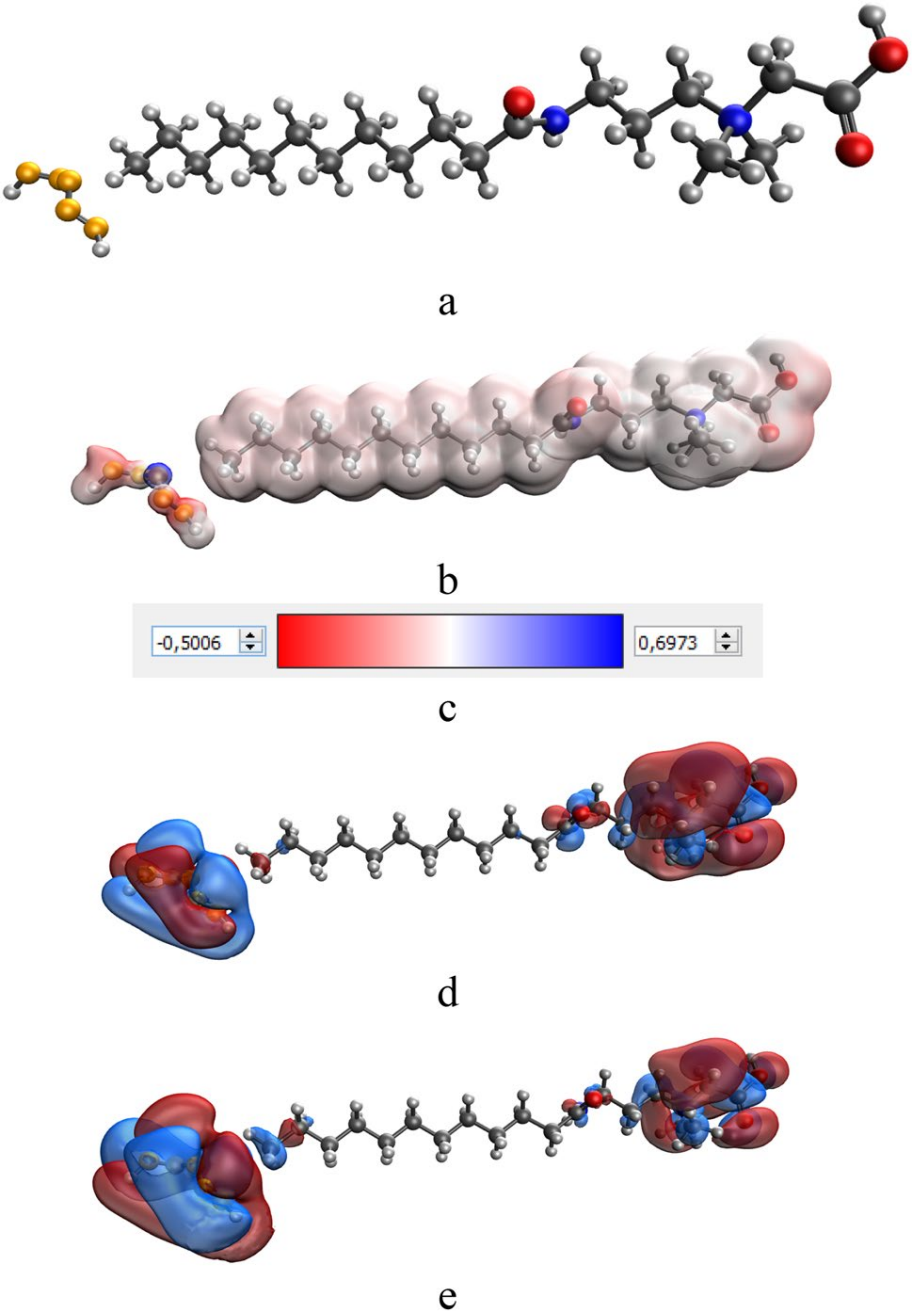
Quantum chemical modeling of the Se-CAPB molecular complex: (**a**) molecular complex model, (**b**) electron density distribution, (**c**) electron density distribution gradient, (**d**) the highest occupied molecular orbital (HOMO), (**e**) the lowest occupied molecular orbital (LUMO).

According to the developed scheme, if the synthesis of Se NPs is carried out in excess CAPB (Fig. 2a), then the surfactant molecule will attach onto the surface of Se NPs with a hydrophobic tail, and the hydrophilic part will be turned to the dispersion medium by positively charged NH^+^ groups. As a result, a positively charged potential-determining layer will form on the surface of Se NPs. The layer of counterions will be formed by oxalic acid anions, which are formed in the reaction system as a result of the oxidation of ascorbic acid. In the case of synthesized Se NPs in excess selenic acid (Fig. 2b), according to the Fajans-Paneth-Hahn Law, the potential-determining layer will be formed due to adsorption of SeO_3_^2−^ anions on the surface of nanoparticles. The counterions layer will consist of CAPB molecules oriented by positively charged NH^+^ groups to the negatively charged surface of Se NPs. To confirm this, we conducted quantum chemical modeling of CAPB molecule and a molecular complex Se-CAPB. The resulting models are shown in Figs. 3 and 4.

Analysis of computer quantum chemical modeling data showed that the total energy of CAPB molecule is − 1082.5 kcal/mol, and the energy of the molecular complex Se-CAPB is − 13074.3 kcal/mol. This fact indicates the energy benefit of the formation process of a chemical bond between Se and CAPB. Our calculations showed that the chemical hardness value (η) of Se-CAPB molecular complex is 0.002. A positive value of this parameter indicates the stability of the complex^41^.

**Figure 5:**
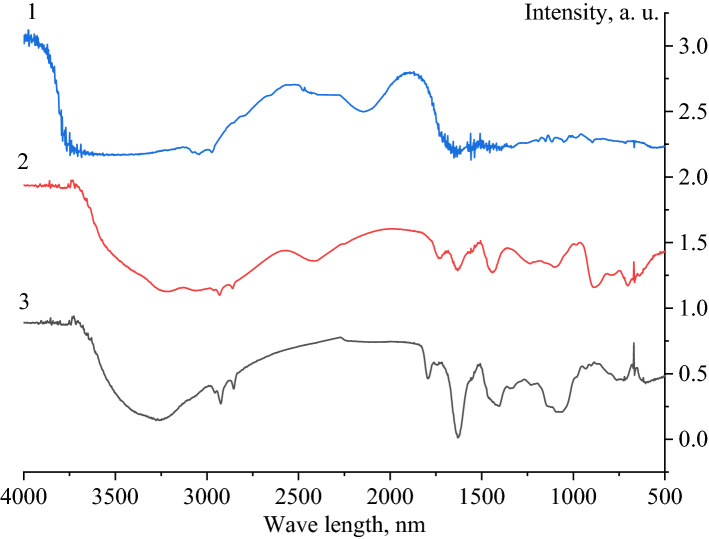
IR spectra of Se NPs samples: 1 – CAPB, 2 – the negative Se NPs sol, 3 – the positive Se NPs sol.

At the next stage, samples of Se NPs were examined by IR spectroscopy. The obtained IR spectra are shown in Fig. 5.

Analysis of the IR spectrum of CAPB showed that in the region from 2800 to 3300 cm^−1^, the presence of oscillation bands characteristic of the CH_2_ group is observed (2854–2924 cm^−1^). In the range from 1300 to 1800 cm^−1^, the presence of single bands of symmetrical oscillations characteristic of the CH_3_ group is observed. These oscillations correspond to the intensity drop areas of 1337 cm^–1^ and 1651 cm^−1^. In the IR spectrum of CAPB there is also a single band of oscillations characteristic of the NH^+^ group corresponding to 1504 cm^−1^. In the range from 1506 to 1584 cm^−1^, there are fluctuations characteristic of the NH^+^ group. In the region from 800 to 1300 cm^−1^, the presence of oscillation bands characteristic of the CH_2_ group is observed (895–986 cm^−1^). In the IR spectrum of CAPB, there are single bands characteristic of the C = O group at 1115 cm^−1^, and the COO^−^ group at 1192 cm^−1^.

Analysis of the IR spectrum of negative Se NPs sol showed that in the region from 2800 to 3300 cm^−1^, the presence of oscillation bands characteristic of the CH_2_ group is observed. Single oscillations characteristic of the CH_3_ group (2955 cm^–1^) and a single oscillation band of the CH group (3252 cm^−1^) are also observed. In the range from 1300 to 1800 cm^−1^, the presence of single symmetrical oscillations characteristic of CH_3_ is observed. These oscillations correspond to the intensity drop areas of 1402 cm^−1^ and 1628 cm^−1^. The oscillation band 1327 cm^−1^ corresponds to the oscillations of the COO^−^ group. In the range from 1516 to 1557 cm^−1^, the presence of oscillation bands characteristic of the NH^+^ group is observed. In the region from 800 to 1300 cm^−1^, there is the presence of bond oscillations characteristic of the CH_2_ group. In the range from 903 to 1060 cm^−1^, there is also the presence of bands of bond oscillations characteristic of C = O group (1130 cm^−1^) and COO^−^ group (1233 cm^−1^).

Analysis of the IR spectrum of positive Se NPs sol showed that in the region from 2800 to 3300 cm^−1^, the presence of oscillation bands characteristic of the CH_2_ group is observed (2853 to 2924 cm^−1^). There are oscillation bands characteristic of CH_3_ group in the range from 2959 to 3052 cm^−1^. The band 3223 cm^−1^ is characteristic of CH group. In the region from 1300 to 1800 cm^−1^, there are bands of symmetrical oscillations characteristic of CH_3_ group. These oscillations correspond to the intensity drop areas in the range from 1630 to 1692 cm^−1^, as well as 1443 cm^−1^. In the region from 800 to 1300 cm^−1^, the presence of CH_2_ group oscillations is observed (885–980 cm^−1^).

Thus, we found that in the IR spectrum of positive Se NPs sol, there is a significant drop in the intensity of bands in the region from 1500 to 1550 cm^−1^, which are characteristic of fluctuations in the bond of the ionized amino group NH^+^, as well as a change in the intensity of bands in the region from 1250 to 1330 cm^−1^ corresponding to fluctuations of COO^−^ group. This fact suggests that CAPB is present in the surface of the particles in the positive Se NPs sol. It is important to note that such changes were not observed in the negative Se NPs sol.

**Figure 6:**
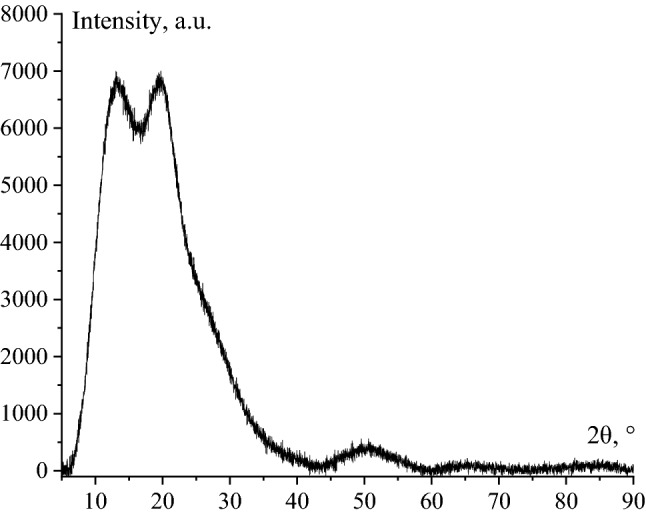
Diffractogram of positive Se NPs sol.

At the next stage of the research, the samples were examined by XRD. One of the obtained diffractograms is shown in Fig. 6.

According to the results of XRD, it was found that Se NPs has a rhombohedral crystal lattice, the space group R-3. The low intensity of the X-Ray characteristic peaks indicates that the structure of the substance is strongly amorphous. An intense broad peak with a maximum of about 22° corresponds to amorphous SiO_2_. The presence of this peak is associated with a feature of sample preparation – usage of aerosil as a substrate for Se NPs.

**Figure 7:**
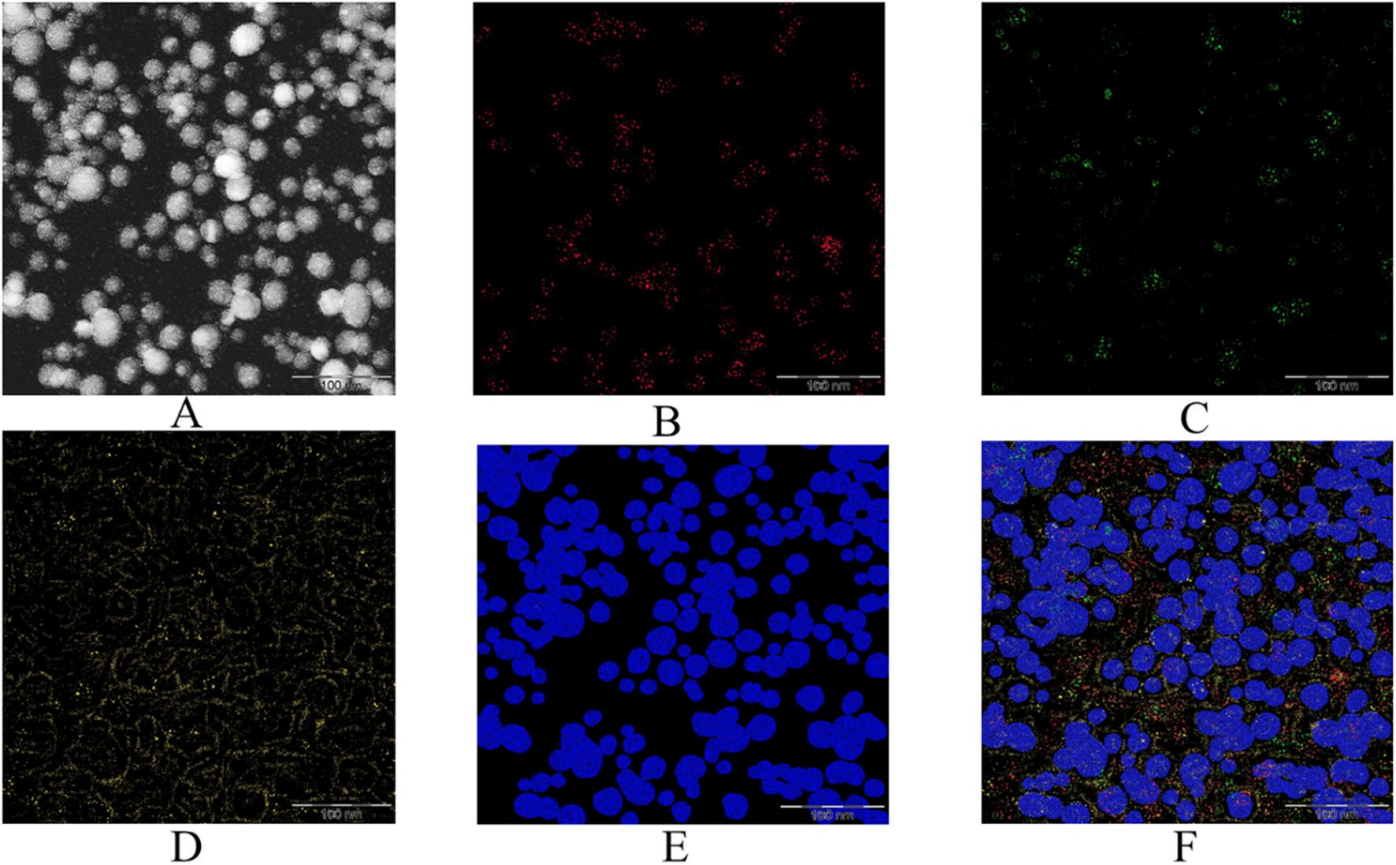
HAADF-TEM image and EDS maps of negative Se NPs sol at magnification × 640000: (**A**) HAADF-TEM image, (**B**) EDS maps of C, (**C**) EDS maps of N, (**D**) EDS maps of O, (**E**) EDS maps of Se, (**F**) EDS maps of all elements.

**Figure 8:**
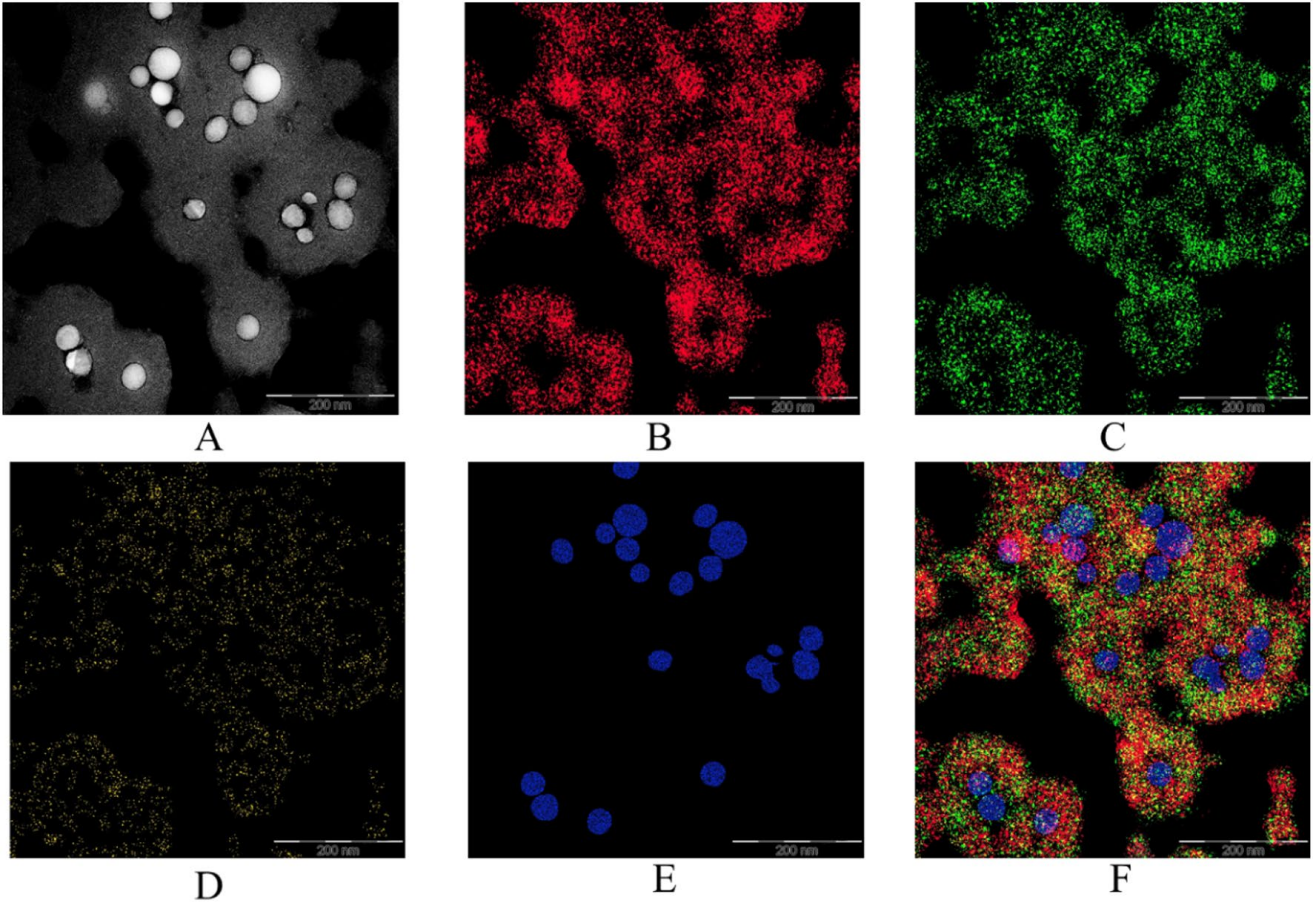
HAADF-TEM image and EDS maps of positive Se NPs sol at magnification × 320000: (**A**) HAADF-TEM image, (**B**) EDS maps of C, (**C)** EDS maps of N, (**D**) EDS maps of O, (**E**) EDS maps of Se, (**F**) EDS maps of all elements.

At the next stage, the samples were examined by the HAADF-TEM method using EDS. The results obtained are presented in Figs. 7 and 8.

The analysis of Figs. 7 and 8 confirmed the above assumptions about the structure of Se NPs. The particle core consists of selenium (Fig. 7E and 8E). In positive Se NPs sol, the C, O and N atoms are uniformly distributed over the shell on the surface of Se NPs, which indicates that this shell is formed by CAPB (Fig. 8A–D, F). In negative Se NPs sol, C and N atoms are located between Se NPs, which indicates that CAPB is not adsorbed on the surface of nanoparticles. However, it is important to note that O atoms are concentrated on the surface of Se NPs, which indicates the presence of selenium oxide formation on the surface of nanoparticles (Fig. 7A–D, F).

### Effect of various ions and active acidity of the medium on the stability of Se NPs

**Figure 9:**
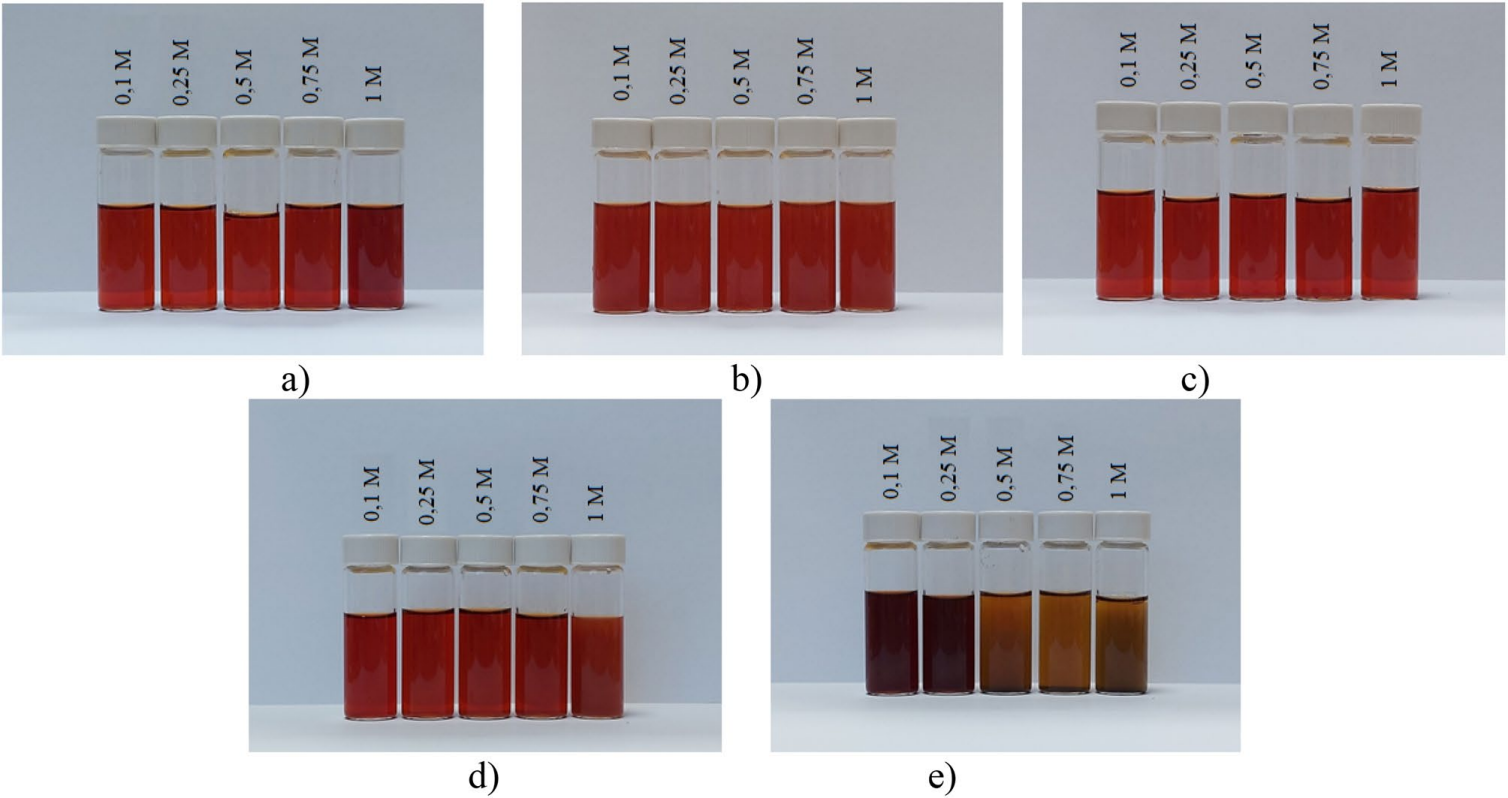
The coagulating effect of salts on the positive Se NPs sol: (**a**) NaCl; (**b**) BaCl_2_; (**c**) FeCl_3_; (**d**) Na_2_SO_4_; (**e**) Na_3_PO_4_.

At this stage, the effect of various ions on the stability of positive and negative Se NPs sols using photon correlation spectroscopy (PCS) and visual assessment of the presence or absence of the coagulation process was investigated. Figure 9 shows images of the positive Se NPs sol with the addition of various salts at concentrations from 0.1 to 1 M. In the process of visual assessment of the samples, it was found that SO_4_^2−^ and PO_4_^3−^ ions had the greatest coagulating effect on the positive Se NPs sol. According to the Schulze-Hardy rule, electrolytes with a large ion charge opposite to the micelle charge have the greatest coagulating ability^42,43^.

**Figure 10:**
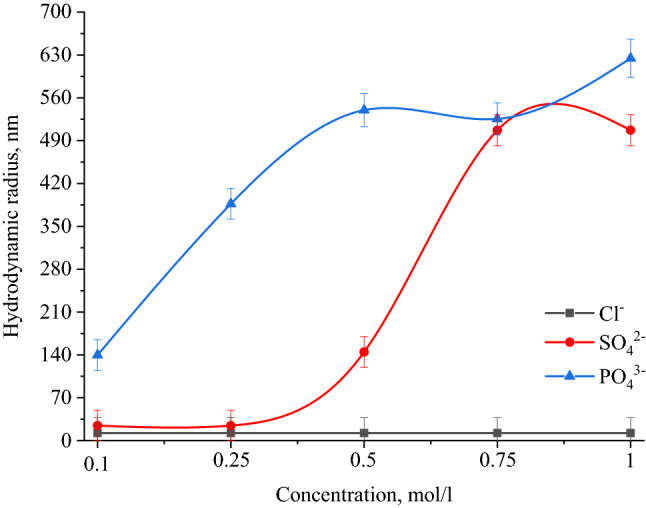
Dependencies of the average hydrodynamic radius of positive Se NPs sol on levels of Cl^−^, SO_4_^2−^ and PO_4_^3−^ ions.

**Figure 11:**
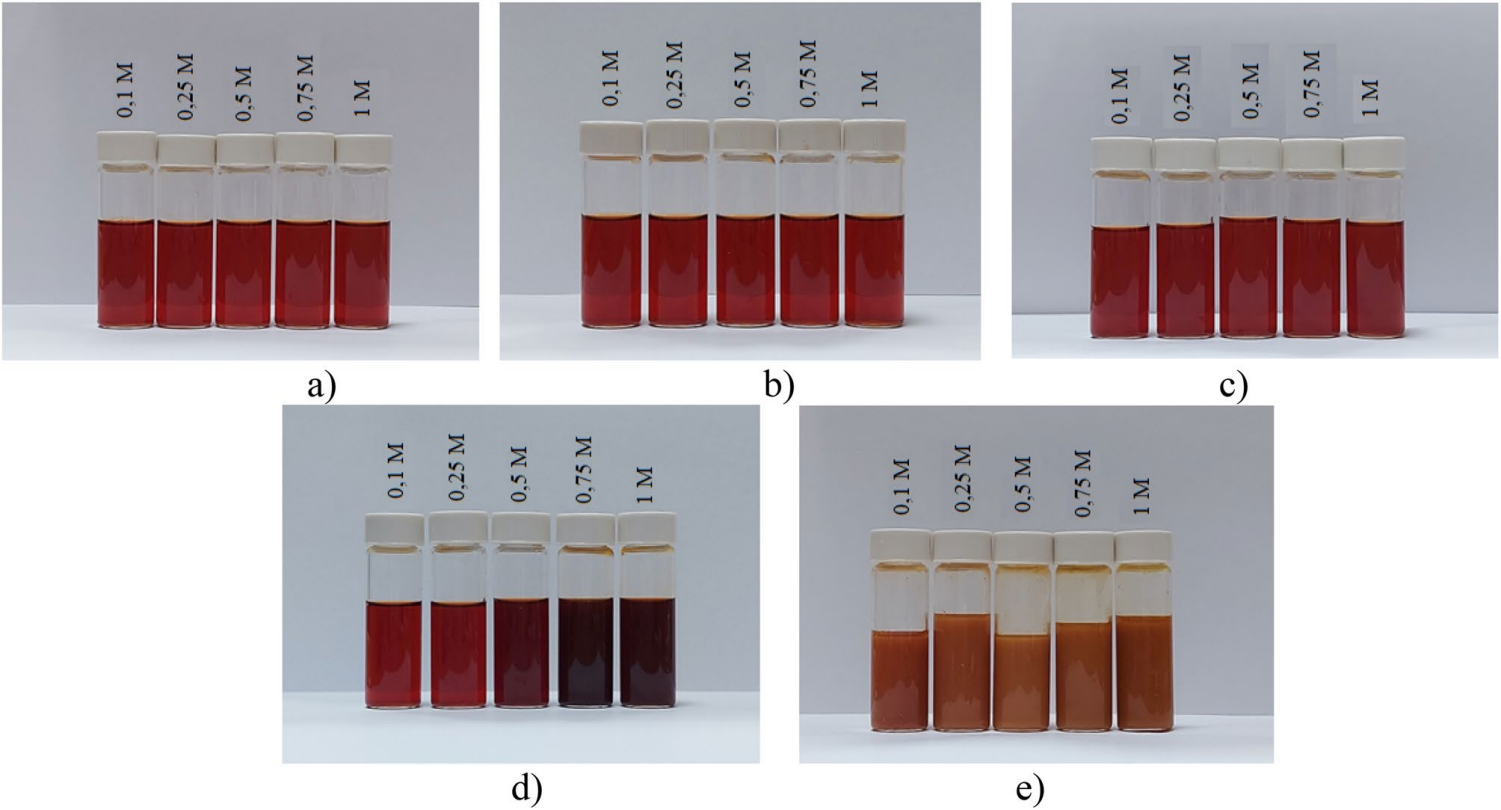
The coagulating effect of salts on negative Se NPs sol: (**a**) *NaCl*; (**b**) *Na*_2_*SO*_4_; (**c**) *Na*_3_*PO*_4_; (**d**) *BaCl*_2_; (**e**) *FeCl*_3_.

Results of PCS showed that when NaCl, BaCl_2_ and FeCl_3_ added to the solution of Se NPs, there were no deviations from the initial results of the average hydrodynamic radius. Figure 10 shows the dependence of average hydrodynamic radius of the positive Se NPs on the concentration of added ions. As can be seen from the dependencies for SO_4_^2−^ and PO_4_^3−^ ions shown in Fig. 10, an increase in the ionic strength of the solution leads to an increase in the size of Se NPs. In terms of the SO_4_^2−^ ion, at the first site, an increase in the concentration of the Na_2_SO_4_ solution to 0.3 M did not lead to a change in the radius of Se NPs and the solutions remained stable. A further increase in the concentration of SO_4_^2−^ ion significantly increased the average hydrodynamic radius of Se NPs. Considering an electrolyte with a greater coagulating capacity – Na_3_PO_4_, the appearance of turbidity in solutions and precipitation due to the coagulation process of Se NPs was observed throughout the concentration range under investigation. At an ion concentration equal to 1 mol/L, the coagulation rate of Se NPs was maximal. Figure 11 shows the dependence of average hydrodynamic radius of the negative Se NPs on the concentration of various ions.

**Figure 12:**
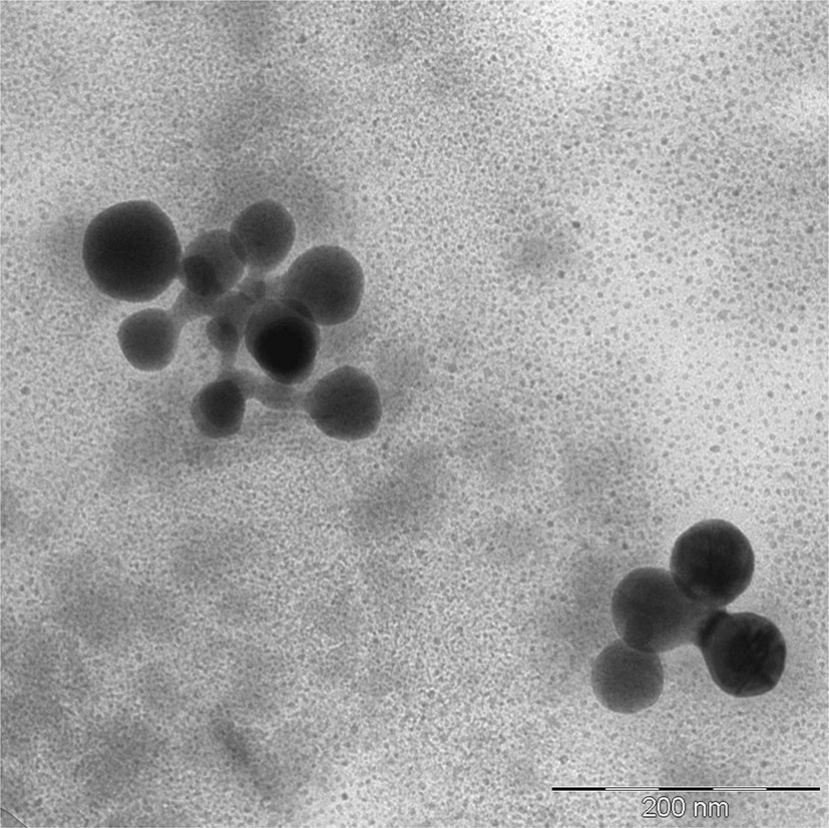
TEM image of a positive Se NPs sol at concentration of Na^+^ 0.25 M (magnification × 320000).

**Figure 13:**
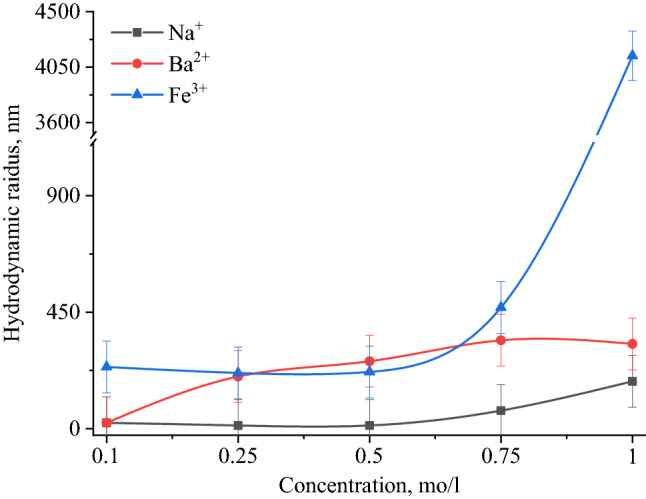
Dependencies of the average hydrodynamic radius of the negative Se NPs sol on the influence of Na^+^, Ba^2+^ and Fe^3+^ ions.

According to the Schulze-Hardy rule, Ba^2+^ and Fe^3+^ cations should have the greatest coagulating effect on the negative Se NPs sol, as can be seen in Fig. 11. This is also confirmed by TEM (Fig. 12) and photon correlation spectroscopy (Fig. 13) that showed the dependence of average hydrodynamic radius of the particles on the ionic strength of the solution.

**Figure 14:**
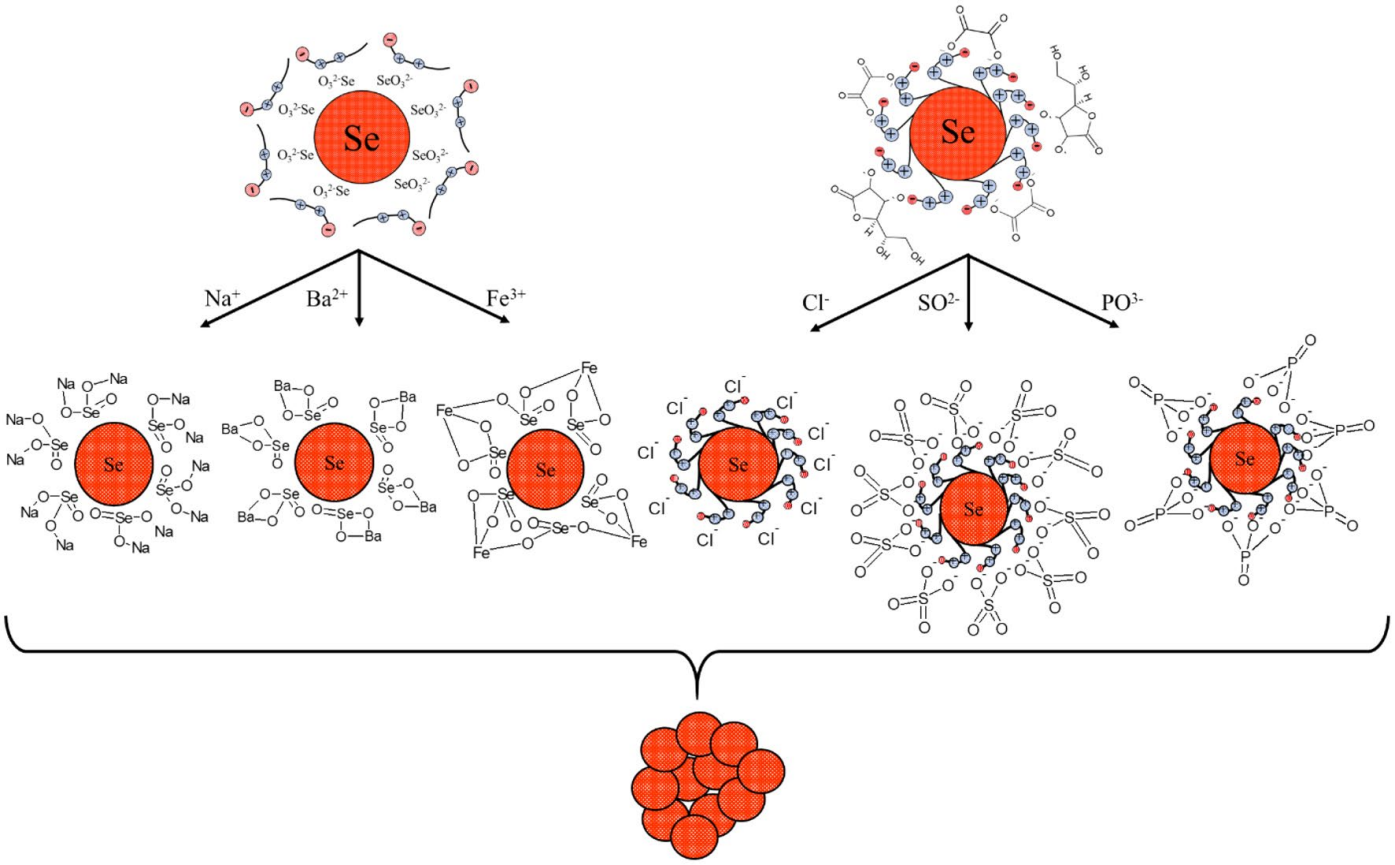
Schematic illustration of coagulation of positive and negative Se NPs sols with different ions.

Higher size of Se NPs occurs with an increase in the concentration of salt in the solution. For Na^+^, there was an increase in the size of colloidal particles from 19 to 200 nm, which is not accompanied by a visual change in sol. For Ba^2+^ ions, we can see a sharp increase in the size of negative Se NPs from 21.67 to 400.3 nm at Ba^2+^ ion levels from 0 to 0.75 mol/L. It is complemented by a visible change in the color of solution, which became cloudy at an ion concentration of 0.75 mol/L. For the salt with the greatest coagulating effect (Fe^3+^), at the lowest level of ions equal to 0.1 mol/L, a visual change in the solution was already observed in the form of a sediment in the solution and also creating a change in color. At higher concentrations of Fe^3+^ there was a sharp increase in size of Se NPs up to 4250 nm. Based on the data obtained, Fig. 14 represents a schematic process of coagulation for the positive and negative Se NPs sols for all the considered salts.

**Figure 15:**
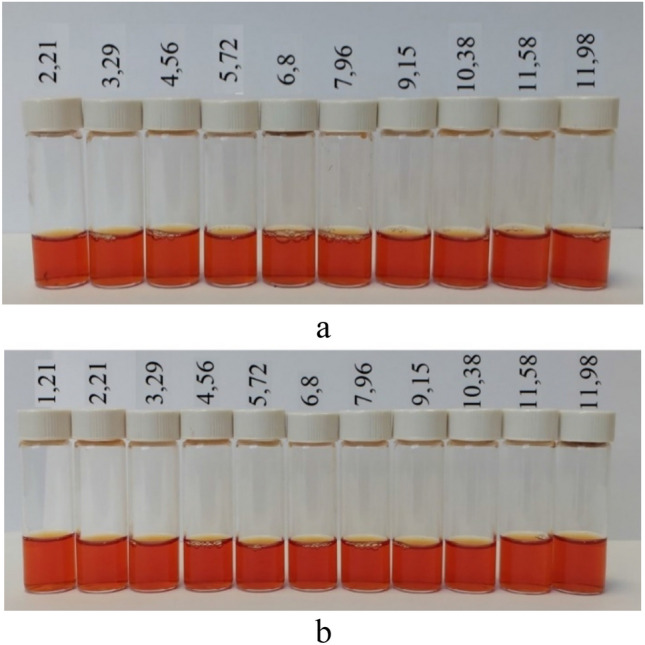
The positive (**a**) and negative (**b**) Se NPs sol at different pH values.

Similarly, the effect of the active acidity of medium on the stability of SeNP solutions was investigated. Figure 15a shows a photo of the positive Se NPs sol at different pH values. As revealed, the effect of different pHs on the stability of SeNP solutions was not visually determined; the solutions remained stable. PCS also didn’t show deviations from the values of average hydrodynamic radii of Se NPs. It also can be seen in Fig. 15b that the pH of medium in the range of 1.81 to 11.98 did not affect the stability of Se NPs. The deviation from the initial values of average hydrodynamic radius of Se NPs can be considered as insignificant.

**Figure 16:**
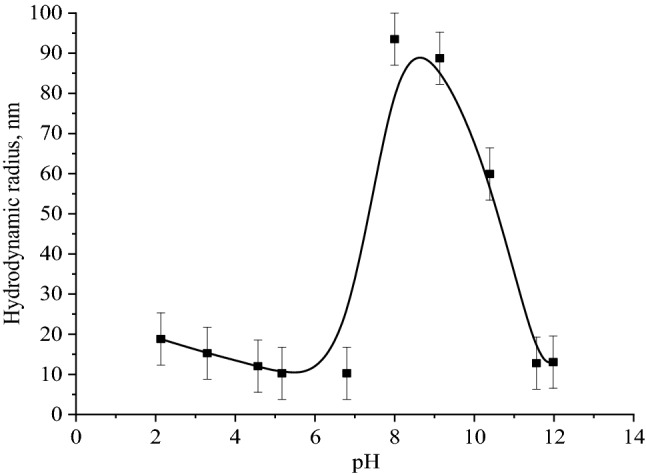
The dependence of average hydrodynamic radius of the positive Se NPs sol on pH after 1 week of exposure.

Since both positive and negative Se NPs sols showed high levels of stability throughout the considered pH range of medium, the solutions were stored for one week, and then the average hydrodynamic radius of Se NPs in the samples was measured. Figure 16 shows the dependence of average hydrodynamic radius of Se NPs on the pH after 1 week of exposure for the positive Se NPs sol. In the pH range from 2 to 7, the average size of Se NPs did not change significantly. In the pH > 7, particle enlargement and coagulation were noted. The maximum hydrodynamic radius was observed at pH = 9.

**Figure 17:**
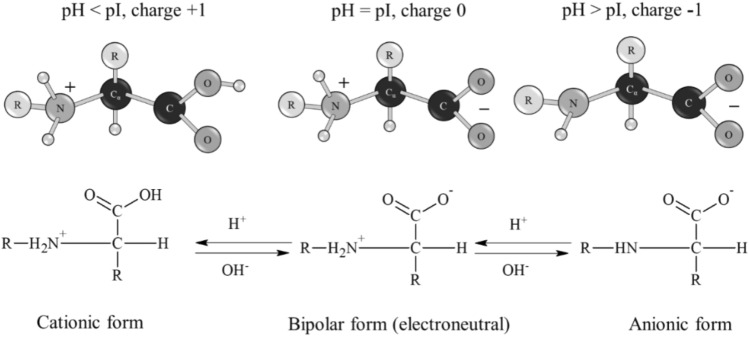
Protonation and deprotonation scheme of the CAPB molecule.

The changes in the size and stability of Se NPs at different pH is because CAPB molecule has both amino and carboxyl groups, which determines its amphiphilic properties ^37^. In an acidic environment, activation of amino groups occurs, and in an alkaline environment, activation of carboxyl groups occurs (Fig. 17. Apparently, when the amino group is protonated in an acidic medium, CAPB molecule acquires a positive charge and attaches this charge to the micelle. With a decrease in the concentration of hydrogen ions (an increase in pH, the speed of protonation process slows down, and the chemical equilibrium shifts in the opposite direction; the charge of the amino groups decreases and becomes zero at the isoelectric point. A further decrease in the concentration of hydrogen ions, above the isoelectric point leads to a change in the charge of CAPB molecule to negative, due to deprotonation of carboxyl groups. The Se-CAPB molecular complex acquiring a negative charge becomes stable.

**Figure 18:**
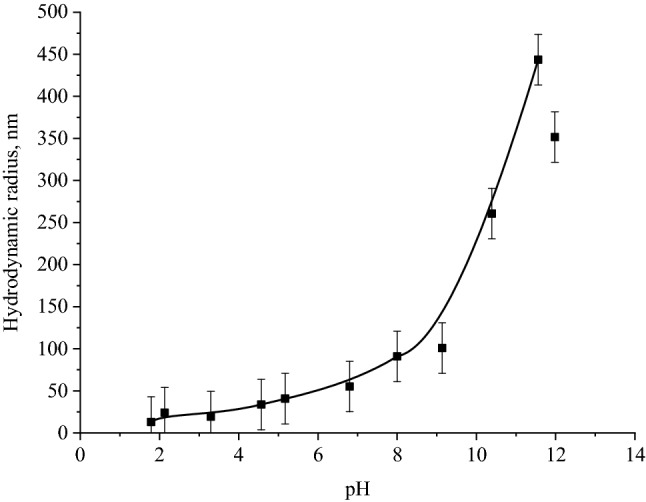
The dependence of average hydrodynamic radius of the negative Se NPs sol on pH after 1 week of exposure.

**Figure 19:**
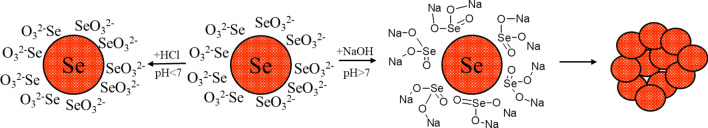
Scheme of coagulation for the negative Se NPs sol.

Figure 18 shows the dependence of average hydrodynamic radius of Se NPs on pH after 1 week of exposure for the negative Se NPs sol. It is radically different from the same dependence for the positive Se NPs sol (Fig. 16). In the pH range from 2 to 9, changes in micelle radii are not significant. An increase in pH > 9 leads to a sharp increase in the size of Se NPs and their coagulation. According to the proposed model for the micelle of the negative Se NPs (Fig. 2b), CAPB molecules are located in the counter ions layer and the diffusion layer. The charge of micelle is determined by selenic acid ions. A variation in pH changes the charge of CAPB from positive in an acidic medium to negative in an alkaline medium. By acquiring a negative charge, the molecules begin to repel negatively charged selenic acid ions and diffuse from the surface of nanoparticles. Selenic acid adsorbed on the surface of Se NPs is neutralized in an alkaline medium, which leads to a loss of stability of the entire system. This process is presented schematically in Fig. 19.

### Assessment of the stability of Se NPs in liquid soap

**Figure 20:**
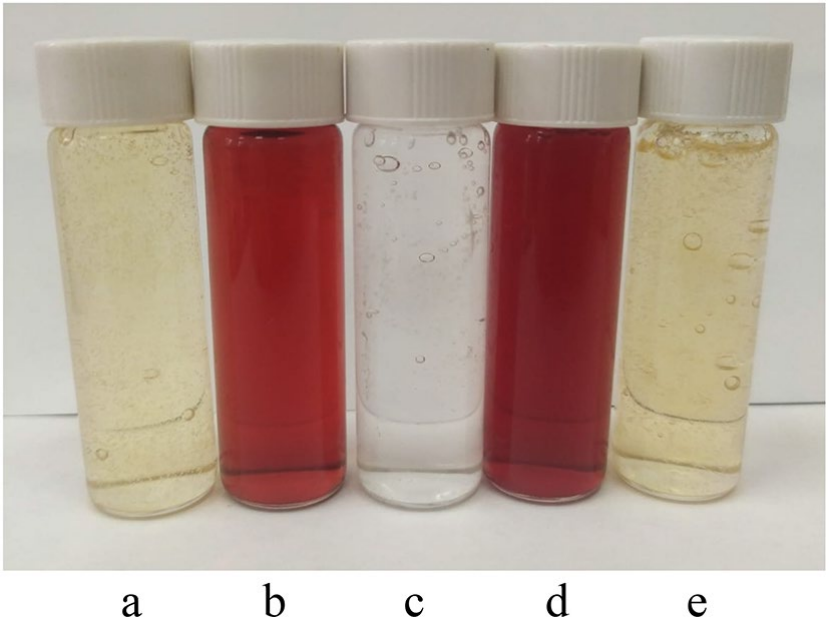
(**a**) liquid soap with the negative Se NPs sol, (**b**) the negative Se NPs sol, (**c**) liquid soap, (**d**) the positive Se NPs sol, (**e**) liquid soap with the positive Se NPs sol.

**Figure 21:**
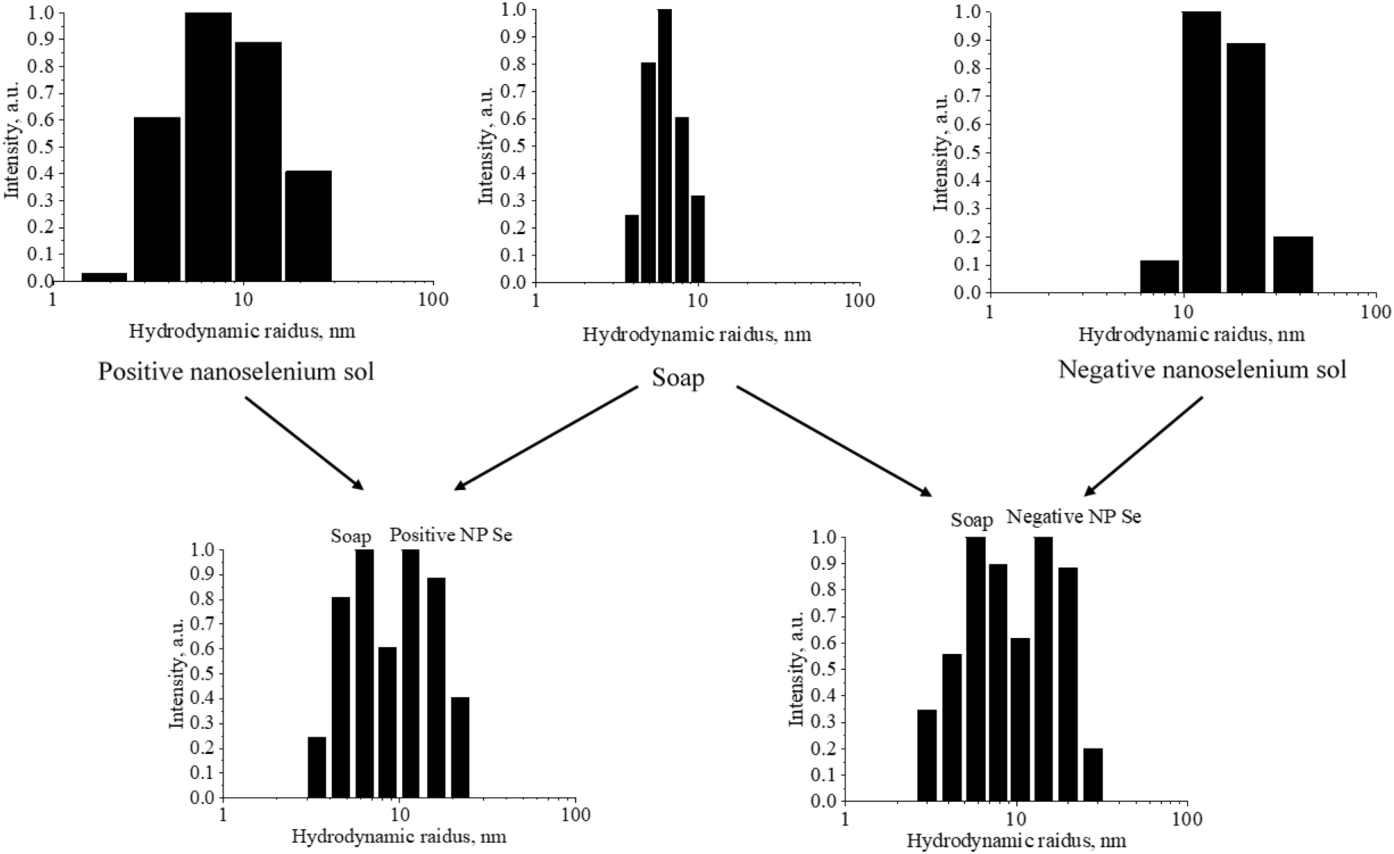
The distribution of hydrodynamic radii of particles in experimental samples of liquid soap.

Industrial application of synthesized Se NPs stabilized with CAPB has a great potential but limited by their stability in products. To assess the stability of Se NPs in real systems, we conducted an experiment in liquid soap (Fig. 20). Adding both negative and positive Se NPs sols to liquid soap changed its color to light yellow. It is important to note that there was no visual turbidity of solutions, which indirectly reveals no coagulation process of Se NPs in liquid soap. In order to confirm this, all samples were studied using PCS. The obtained histograms for the distribution of hydrodynamic radii are shown in Fig. 21.

Figure 21 shows that the average hydrodynamic radius of Se NPs was 6–7 nm for the dispersed phase of liquid soap, 10 nm for the positive Se NPs sol and 20 nm for the negative Se NPs sol. The liquid soap samples with positive or negative Se NPs had two phases: dispersed phase of liquid soap and Se NPs. At the same time, the particles of these phases retained their initial distributions, which reveals the stability of Se NPs in the liquid soap medium.

At the final stage of the experiment, physicochemical characteristics of the liquid soap samples were studied (Table 1). It can be concluded that the positive and negative Se NP sols did not significantly affect the physicochemical properties of the liquid soap and can be considered for introduction into the technological cycle of liquid soap production.

**Table 1:** Physicochemical properties of experimental samples of liquid soap.

Physico-chemical properties	Liquid soap	Liquid soap with the positive Se NPs sol	Liquid soap with the negative Se NPs sol
pH	6.8 ± 0.03	7.0 ± 0.03	6.9 ± 0.03
Dynamic viscosity, mPa·s	1769 ± 10	1751 ± 10	1763 ± 10
Electrical conductivity of 5% solution, mS	2212.1 ± 1.3	2120.4 ± 1.3	2217.1 ± 1.3
Surface tension of 5% solution, N/m	31.5 ± 0.1	30.2 ± 0.1	31.1 ± 0.1
Refractive index	1.4215 ± 0.0002	1.4216 ± 0.0002	1.4216 ± 0.0002

## Conclusion

Within the framework of the presented study, Se NPs stabilized with CAPB were synthesized by chemical reduction in an aqueous medium. The resulting nanoparticles were characterized by a spherical shape with an average size of about 20–30 nm and 40–50 nm for positive and negative sols, respectively. When studying the stability, it was found that the stability of positive Se NPs was influenced by SO_4_^2−^ and PO_4_^3−^ ions, and the stability of negative Se NPs was influenced by Ba^2+^ and Fe^3+^ ions, which is in line with the Schulze-Hardy rule. The influence of pH on the stability revealed that the positive and negative Se NPs sols had high levels of stability in the considered range of pH from 1.21 to 11.98. Finally, the experimental samples of liquid soap with the positive or negative Se NPs had particle phases that characterized the particles of the dispersed phase of liquid soap and Se NPs. The particles of these phases retained their initial distributions, which confirmed the stability of Se NPs in the liquid soap medium.

The data obtained revealed the wide possibilities of practical application of Se NPs stabilized with CAPB in various products of the perfumery, cosmetic, pharmaceutical, food and agricultural industries. In this connection, at the next stage of research, we plan to study the antioxidant activity, hypoallergenic properties, toxicological characteristics of Se NPs stabilized with CAPB, as well as their transdermal transfer and antitumor activity. Moreover, given the natural antimicrobial effect of CAPB, as well as the promising antimicrobial potential of Se NPs, the resulting nanohybrid systems can cause a promising synergistic effect in the production of antiseptics and disinfectants, which is especially relevant in the current situation in the world with COVID-19. Therefore, the study of bactericidal, fungicidal and antiviral properties of the developed molecular complexes is also a priority for future research.

## Data Availability

The data that support the findings of this study are available from corresponding authors upon request.
